# Advances in Basivertebral Nerve Ablation for Chronic Low Back Pain: A Narrative Review

**DOI:** 10.3390/jpm15030119

**Published:** 2025-03-19

**Authors:** Sujeivan Mahendram, Paul J. Christo

**Affiliations:** Division of Pain Medicine, Department of Anesthesiology and Critical Care Medicine, The Johns Hopkins University School of Medicine, Baltimore, MD 21205, USA; smahend1@jh.edu

**Keywords:** basivertebral nerve ablation, vertebrogenic, axial low back pain, Modic changes

## Abstract

Chronic low back pain has traditionally been thought to stem from intervertebral disc degeneration. However, emerging evidence over the last few decades has revealed other contributing sources. One such etiology of chronic non-radiating axial low back pain has been attributed to vertebral end plate disruption and degeneration, leading to basivertebral nerve-mediated nociception. These degenerative events, described as Modic changes on MRI, provide a means of diagnosis and offer personalized treatment options, like minimally invasive radiofrequency ablation, to help address this source of low back pain. This review focuses on recent advancements, rationale, efficacy, and safety profile intraosseous basivertebral nerve ablation in the treatment of vertebrogenic back pain, and discusses current knowledge gaps that may help guide future research in the field.

## 1. Introduction

Chronic low back pain (CLBP) is one of the leading causes of worldwide disability as well as one of the most expensive causes of work absenteeism in individuals between 30 and 64 years of age [1,2,3,4,5]. In fact, it is the most common occupational disorder in the US with an estimated prevalence of 60–85% over the course of one’s lifetime [6,7]. This particular subset of CLBP, formally defined as axial low back pain lasting more than three months, can further be difficult to elucidate due to various pain generators that may be involved. This can include, but is not limited to, facetogenic, discogenic, or a vertebrogenic source of CLBP. Further classifying CLBP into anterior and posterior column-derived pain can assist in making an accurate diagnosis.

**Figure 1:**
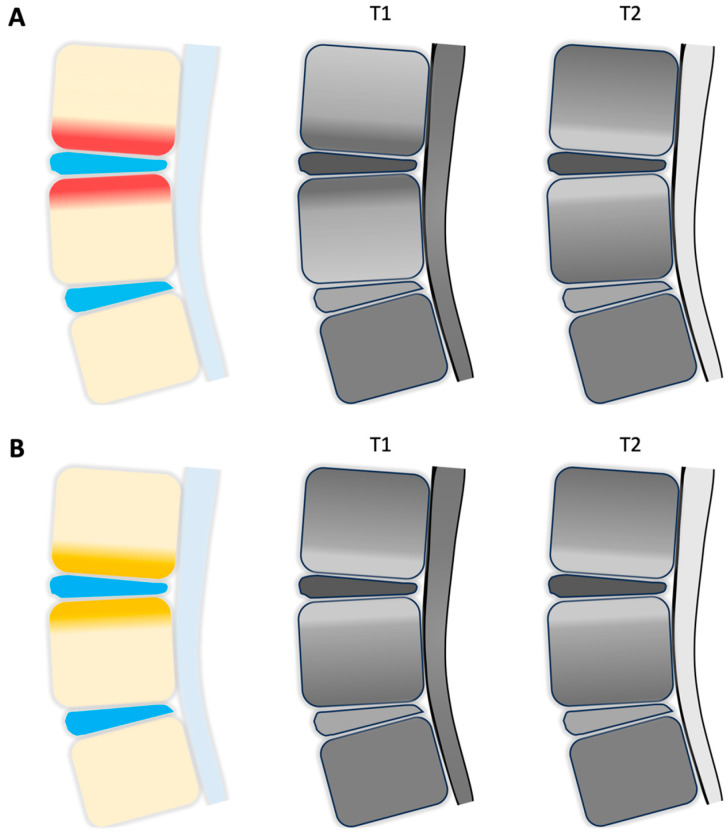
Schematic representation of Modic Changes on T1- and T2-weighted MRI. (**A**) Modic Type 1 changes as a result of endplate inflammation (red) presenting as hyperintensity on T1 and hypo-intensity on T2 imaging. (**B**) Modic Type 2 changes reflective of endplate fatty infiltration (yellow) illustrating hypo-intensity in both T1 and T2 imaging.

Vertebrogenic back pain is characterized by axial pain that originates from the vertebral endplates of degenerative discs. Nociception is attributed to activation of the basivertebral nerve (BVN), which originates from the sinuvertebral nerve (SVN) and enters the posterior vertebral body via the basivertebral foramen. The BVN then terminally branches to innervate the superior and inferior endplates [8,9]. Vertebral endplate alterations, commonly associated with Modic changes on T1 and T2 weighted magnetic resonance imaging (MRI), can be helpful in determining a diagnosis (see Figure 1). For instance, Modic type 1 changes are suggestive of endplate inflammation, which are demonstrated by T1 hypo-intensity and T2 hyperintensity on MRI imaging. Modic type 2 changes are indicative of fatty infiltration and seen as hyperintensity on both T1 and T2 weighted MRI. Lastly, Modic type 3 changes are seen as hypo-intensity on both T1 and T2 weighted MRI and are suggestive of bony sclerosis. Though the presence of both type 1 and 2 Modic changes largely support a diagnosis of vertebrogenic back pain, Type 3 Modic changes do not, as they are associated with sclerotic changes [10]. Traditional treatments for vertebrogenic back pain, including physical therapy, medical management, and invasive surgical interventions, often provide only limited and temporary relief. Given that the BVN is a key pain generator of anterior axial back pain, the emergence of minimally invasive BVN ablation (BVNA), also known as the Intracept procedure, during recent years has demonstrated its early safety profile and efficacy in treating this source of low back pain. By using radiofrequency, BVNA can effectively disrupt nociceptive signaling originating from endplate disruption. Currently, Intracept is approved by the US Food and Drug Administration (FDA) for patients with at least 6 months of CLBP who have not responded to at least 6 months of conservative management, and who display Modic type 1 or 2 changes between vertebral levels L3 through S1.

Furthermore, while techniques such as medial branch joint radiofrequency ablation (RFA) can be helpful in addressing posterior axial back pain due to facet joint disease, they do not reach the BVN and, therefore, do not target anterior axial low back pain. Here, we review recent advancements in BVNA and its utility in treating vertebrogenic-mediated CLBP, thus providing a more personalized approach to pain medicine when managing such patients.

## 2. Materials and Methods

This study was conducted in accordance with the five elements commonly discussed in narratives reviews: (1) rationale for review; (2) clarity of boundaries, scope, and definitions; (3) justification for inclusion and exclusion criteria; (4) reflexivity and saturation/sufficiency statement; and (5) details of analysis and interpretation [11]. Database sources included PubMed, Medline, and Google Scholar.

### 2.1. Inclusion Criteria

All human studies in the English language performed in the past 20 years (December 2014–December 2024) assessing basivertebral nerve ablation for the treatment of chronic low back pain were included in the database search criteria.

### 2.2. Exclusion Criteria

All case reports, letters, book chapters, preprints, and articles without patient-related outcomes were excluded from this review.

## 3. Results

**Figure 2:**
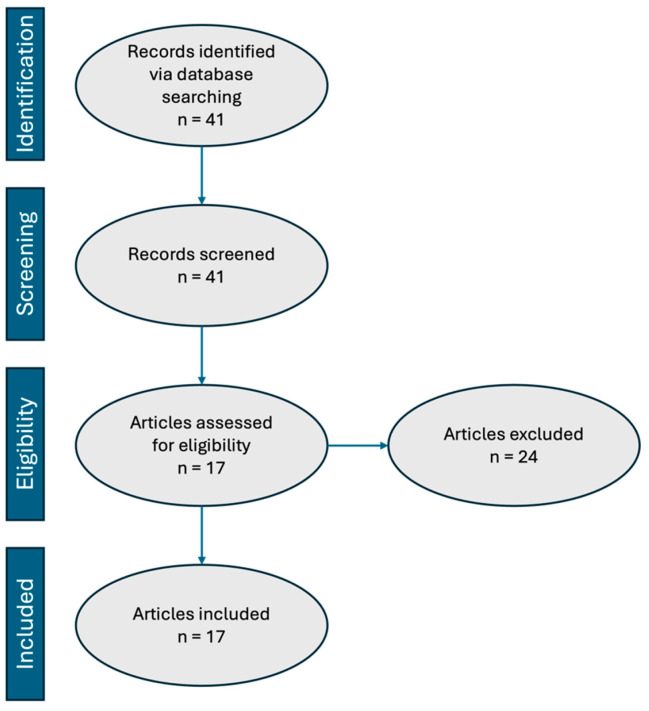
PRISMA flow diagram. Adapted from Page et al., 2020 [12].

A total of 41 articles were identified based on search criteria across all three databases. Among these, there were 1 case report, 4 letters to the editor, 1 research letter, 1 commentary, 3 systematic reviews, 7 reviews on CLBP citing BVNA, 2 studies that reviewed relevant anatomy, and 3 studies that described procedural technique. Additionally, 2 studies were excluded due to methodology discordance because they presented data targeting both BVN and SVN. All of the aforementioned publications were excluded, as they did not present patient-related outcomes, thus leaving 17 articles available for review. Figure 2 summarizes the sequential steps taken according to the preferred reporting items for systematic review and meta-analyses (PRISMA) guidelines. All included articles were organized chronologically by publication date and are presented in Table 1.

## 4. Discussion

### 4.1. Efficacy

BVNA has emerged as an effective tool to help treat patients with vertebrogenic CLBP. Several clinical studies demonstrate the efficacy of BVNA in improving functional disability and reducing pain scores. The majority of studies reviewed report the Oswestry Disability Index (ODI) and Visual Analog Scale (VAS) as primary outcomes.

The first pilot study using Intracept technology was published by Becker et al. (2017). In their prospective study, which evaluated patients with CLBP unresponsive to conservative treatment, ODI scores significantly decreased following intervention at 6 weeks, 3 months, 6 months, and 12 months follow-up periods. They further reported significant improvements in VAS scores at 6 weeks and 3 months. However, this particular study did not include a control group and thus definitive conclusions could not be made [13].

The SMART Trial by Fischgrund et al. (2018) was a prospective randomized double-blind sham-controlled multi-center study that included patients with CLBP (≥6 months) who did not respond to non-operative management for at least 6 months. Here, they reported a significant reduction in ODI scores at 3- and 12-month post-procedure [14]. At the one-year time point, patients in the sham procedure arm were offered crossover into active treatment, which transitioned this study into a single-arm prospective trial. By 24 months, both ODI (53.7%) and VAS (52.9%) scores were also shown to have significantly improved from baseline [9]. Furthermore, among the 117 treated patients, 100 were available for follow-up more than 5 years post-procedure, which revealed a significant improvement in ODI (mean reduction of 26 points) and VAS (mean reduction of 4.38) scores compared to baseline [19]. Collectively, these studies demonstrated that BVNA provides clinically meaningful improvements in pain and function for at least 5 years.

Truumees et al. (2019) conducted a prospective single arm open label study with the aim of broadening the inclusion criteria (i.e., use of opioid medication and history of lumbar laminectomy or discectomy) compared to the previously reported randomized controlled trials. Though the most commonly targeted level was L5–S1 (71%), there was no mention as to whether the procedure was performed at the levels of prior laminectomies. An interim analysis of the first 28 treated patients with vertebrogenic-related CLBP between L3 and S1 vertebral levels showed a clinically and statistically significant benefit in pain (≥2 cm decrease in VAS in 75% of patients) and function (> 10 point improvement in ODI scores in 93% of patients) at 3 months post-procedure [17]. Subsequently, Macadaeg et al. (2020) published a similar study evaluating 48 patients from a community practice setting. They similarly demonstrated that, by 12 months, there was a significant decrease in both ODI (89% reported > 15 point decrease) and VAS (69% reported 50% reduction on pain scale) scores [20].

The INTRACEPT Trial was a prospective open label randomized controlled multicenter trial that compared BVNA to standard of care. In this study, conventional therapy consisted of pain medications, physical therapy, and spinal injections [16,21,22]. An interim analysis at 3 months post-procedure demonstrated a significant improvement in ODI (−25.3 points) and VAS (−3.46 points) scores from baseline [16]. At the conclusion of the extended 12 month study, Smuck et al. (2021) reported similar clinically significant findings at 3-, 6-, and 12-month follow-ups [21]. Furthermore, Koreckij et al. (2021) subsequently reported similar results for the BVNA arm at 24 months (also by extension of the INTRACEPT Trial), which demonstrated that ODI and VAS improved by 28.5 ± 16.2 points and 4.1 ± 2.7 cm, respectively [22].

The majority of studies reviewed used highly specific inclusion and exclusion criteria during patient selection. In particular, Modic Type 1 and 2 changes remained the most applicable and useful criteria for determining BVNA candidacy. For instance, in their aggregated cohort of three prospective clinical trials, McCormick et al. (2022) demonstrated that baseline MRI characteristics, such as the presence of anterior and posterior column degeneration, did not reveal any clinically significant association with BVNA success [24]. In the same year, McCormick et al. used a similar study design to investigate the relationship between pain location and exacerbating activities with treatment success following BVNA. Their study showed that midline low back pain correlated well with successful treatment at 3 months follow-up. Factors that were associated with increased odds of treatment success included pain duration > 5 years, low back pain exacerbation with activity and spinal extension, lack of epidural steroid injection (6 months prior to BVNA), and lack of opioid use [26]. A similar study by Boody et al. (2022) assessing demographic and clinical characteristics of patients undergoing BVNA showed that pain duration >5 years and higher baseline ODI scores increased the odds of successful treatment, while baseline opioid use and higher Beck Depression Inventory (BDI) scores reduced these odds. However, the regression models used by Boody et al. demonstrated a receiver operating characteristic (ROC) of <70% areas under the curve, which by definition limited the overall predictive value of the intervention [25].

Given that BVNA is a relatively new intervention, the long-term effects on spinal biomechanics and the potential need for repeat procedure remain areas of active investigation. There is emerging clinical evidence in support of BVNA (compared to conventional therapy) that suggests long-term reduction in pain score and functional improvement, with follow up data extending to five years. Recently, Smuck et al. (2023) reported the 3-year outcomes from the BVNA treatment arm aggregated from two prospective trials. Their findings revealed statistically significant improvements in mean ODI (−31.2 ± 13.6) and numerical pain score (−4.3 ± 2.3), as well as a 74% reduction in opioid use and 84% reduction in the use of spinal interventions at 3 years [27]. Furthermore, in their pooled cohort analysis of three clinical trials, McCormick et al. (2024) demonstrated clinically and statistically significant reductions in low back pain-related healthcare utilization. Specifically, they illustrated that there was a significant decrease at 1- and 5-years following BVNA treatment of both opioid use (40.3% and 61.7%, respectively) and therapeutic lumbosacral injections (66.5% and 65.3%, respectively) [28]. This duration of relief especially distinguished BVNA from other therapies, such as epidural steroid injections, which often provide shorter-term relief of patient symptoms.

Among the 17 reviewed studies, only two were conducted independently of industry funding. First, Kim et al. (2018) published a retrospective observational study, in which they used a transforaminal epiduroscopic basivertebral nerve laser ablation (TEBLA) to treat CLBP [15]. In their investigation, 14 patients with a history of at least 6 months of CLBP, who were unresponsive to at least 4 months of conservative treatment, were enrolled. Patients were also screened to include the presence of Modic type 1 or 2 changes based on MRI and provocative discography. VAS scores (baseline 7.8 ± 1) decreased significantly at the 1-week (1.9 ± 1.4), 3-month (2.2 ± 0.9), and final (12–20 months; 2.4 ± 1) follow-up periods. Although the investigators did not assess function, they reported patient satisfaction using MacNab’s criteria, which revealed 50% of patients reporting excellent satisfaction, and 43% good. Moreover, this study uniquely analyzed target levels that extended from L2 to S1. The second study, by De Vivo et al. (2020), consisted of a prospective experimental uncontrolled trial that placed an emphasis on the need for other imaging modalities, like single photon emission computed tomography (SPECT-CT), rather than just the presence of Modic type 1 or 2 changes during the patient selection process [18]. For example, the investigators performed CT-guided BVNA ablation in 56 patients using an articulating bipolar radiofrequency electrode (STAR Tumor Ablation System, Merit Medical). Clinical success of both VAS (defined as > −2.0 cm from baseline) and ODI (defined as > −10 points) was achieved in 54/56 patients at the 12-month follow up. These findings were comparable to the majority of previously published data on fluoroscopic-guided procedures. Taken together, neither of these studies reported any serious adverse effects.

Overall, our review of the literature is consistent with the conclusions of a recent systematic review suggesting moderate-quality evidence of BVNA for improving pain and function in select patients with CLBP compared to conventional therapy or sham procedure [29].

### 4.2. Safety Profile

Regardless of selection criteria, general contraindications to BVNA include active infection, pregnancy, incomplete skeletal maturity, previous spinal surgery at the target site, type 3 Modic changes, and the presence of pre-existing implantable pulse generators, because the procedure could interfere with device function [9,16,17,30]. Nonetheless, this minimally invasive technique has been associated with low complication rates [14,16,17,20,21]. Among all reviewed studies, there were no device- or procedure-related serious adverse events, such as thermal injuries. However, adverse events that have been reported include transient motor/sensory deficits, incisional pain, nerve root injury, lumbar radiculopathy, and urinary retention. One study reported a single instance of vertebral compression fracture in an osteopenic patient (sham arm); however, the fracture healed by 8 weeks without further complication [14]. The same study reported one case of retroperitoneal hemorrhage due to misplaced trocar placement at the pedicle. MRI evaluation at 6 weeks and 6 months following the procedure, however, showed no evidence of accelerated disc degeneration, spinal cord abnormalities, or avascular necrosis [14]. Nevertheless, only a handful of studies published to date discuss adverse events and complication rates.

It is important to note that the safety profile to date is based on several prospective randomized controlled trials that exclude older adult patients with other spinal diagnoses, including scoliosis and osteoporosis. Given that BVNA has expanded to patients beyond those included in randomized controlled trials, the inclusion of patients with co-existing spinal diagnoses, such as osteoporosis, has highlighted an increased incidence of low energy vertebral compression fractures as a new source of back pain approximately 2 months following BVNA treatment [31]. For example, Fogel et al. (2023) [31] performed BVNA on 74 patients, in whom 8 developed low energy vertebral compression fractures. The mean age of the fracture group was 78 years, which represented 11% of the study group, and reflected a much higher fracture rate compared to the 0.4% prevalence of vertebral compression fractures that has been reported in previous clinical trials [13,14,16,17]. It may be valuable for older adults at risk of bone demineralization to undergo bone mineral density testing prior to BVNA treatment in order to stratify them based on level of risk of developing vertebral compression fractures. Given that the majority of trials to date report data based on a mean age of 40–50 years, further research in the field will need to address the appropriateness of conducting BVNA in the older adult population.

### 4.3. Limitations

First, patient selection is an essential component to ensuring desired outcomes, since the procedure is most beneficial in individuals with vertebrogenic CLBP and Modic Type 1 or 2 changes. Given that this procedure targets a specific pain generator along the vertebral endplates, it will not benefit patients with CLBP arising from other pain sources, such as the facet joints. The strict selection criteria used in the majority of reviewed studies results in a very specific patient population that can benefit from this treatment. Recognizing that stringent criteria are important for testing new devices in clinical trials, the results may not be generalizable to real-world applications of BVNA. Further investigations, such as that of Truumees et al. (2019) [17], which broaden selection criteria are warranted in order to be able to assess generalizability to older adults and those who have undergone spinal surgical procedures, for instance.

Secondly, a large number of the ‘landmark’ papers in the field are industry-funded, which represents a potential source of publication and selection bias for the data being presented. For example, there is evidence that industry-sponsored studies may report lower complication rates and higher rates of positive outcomes [32,33]. Given that all but two studies reviewed were industry-sponsored, future non-industry supported studies would assist in confirming reported outcome data. Furthermore, studies examining long-term efficacy will help establish the durability of the procedure given that most of the data currently exists for short-term outcomes.

Although BVNA is a relatively novel technique, alternative ablative methods have recently been developed to target the BVN. For instance, the TEBLA technique involves the application of yttrium aluminum garnet (YAG) to ablate the BVN terminus via the supra-pedicular notch [15]. An epiduroscopic bipolar RFA of the BVN and SVN has also been described [34]. The utility of other minimally invasive techniques, including intradiscal RFA and intradiscal methylene blue, have significantly declined due to inconsistent clinical outcomes and lack of strong evidence supporting long-term efficacy.

## 5. Conclusions and Future Directions

In summary, BVNA has significantly advanced the field of precision pain medicine by offering an opioid-sparing treatment option tailored towards a unique subset of individuals with CLBP. Specifically, it offers a minimally invasive approach for patients with axial vertebrogenic CLBP lasting more than 6 months, failing at least 6 months of conservative therapy, and with evidence of Modic type 1 and 2 changes. Its ability to specifically treat low back pain of vertebrogenic origin, while providing a means of multimodal pain management, further aligns with the principles of personalized medicine. As we continue to refine patient selection criteria and gather more information from long-term data, BVNA is poised to rank higher in the treatment algorithm for the multidisciplinary management of CLBP.

New applications of BVNA are also worth noting. For instance, hypersensitive BVN pathology leading to lumbar muscular spasms has previously been shown to be associated with epidural neovascularization with adhesion [34]. In one particular study, the operators identified a unique intraoperative buttock and paraspinal muscle twitching, referred to as “Kim’s twitching,” during BVN and SVN ablation procedures. The study group showed a high association of the presence of Kim’s twitching and high-grade neovascularization with hypersensitive nerves, in addition to having favorable outcomes following RFA. This neovascularization with adhesion is believed to be a result of aberrant neural connections, which are associated with a reflex inhibitory mechanism of the multifidus muscle, ultimately inducing spasms. Therefore, BVNA may effectively alleviate such paraspinal muscle spasms in patients with lumbar degenerative disc disease, particularly those with epidural neovascularization with adhesion [35].

Based on the current review of available literature, the evidence supports BVNA as both efficacious and safe in appropriately selected patients. Given that BVNA using Intracept is only approved for pathology occurring from L3 to S1, it would be instructive to determine whether the outcomes related to safety, efficacy, and adverse events would remain unchanged at higher vertebral levels. Future investigations might focus on the value of treating vertebrogenic cervical and thoracic pain, the risks of treating these levels, and the type of equipment modifications required to address diverse spinal levels. Ultimately, the evidence suggests that BVNA provides a personalized pain treatment modality that improves function, quality of life, and patient satisfaction.

## Figures and Tables

**Table 1:** Summary of Included Studies.

Author	Year	Journal	Design	Number of Participants	Follow-Up	Primary Outcome	Clinical Significance	Comments
Becker et al. [13]	2017	The Spine Journal	Prospective, single arm	16	6 weeks; 3, 6, 12 months	Pain score (VAS), ODI	Reduction in VAS, ODI scores	Transpedicular and extra-pedicular approachesIndustry sponsored
Fischgrund et al. [14]	2018	European Spine Journal	Prospective randomized double-blind sham-controlled multi-center	225	2, 6 weeks; 3, 6, 12, 24 months	ODI	Reduction in ODI scores	Treatment (*n* = 147) Sham (*n* = 78) Industry sponsored
Kim et al. [15]	2018	Pain Research and Management	Retrospective, observational single-center	14	12–20 months	Pain score (VAS, MacNab)	Reduction in VAS, MacNab scores	Transforaminal epiduroscopic approach
Fischgrund et al. [9]	2019	International Journal of Spine Surgery	Prospective randomized double-blind sham-controlled multi-center	147	2, 6 weeks; 3, 6, 12, 18, 24 months	Pain score (VAS), ODI	Reduction in ODI, VAS scores	Industry sponsored
Khalil et al. [16]	2019	The Spine Journal	Prospective open label randomized controlled trial	140	3, 6, 9, 12 months	ODI	Reduction in ODI scores	BVNA (*n* = 51) Standard care (*n* = 53) Industry sponsored
Truumees et al. [17]	2019	European Spine Journal	Prospective open label single arm multi-center case series	28	3 months	ODI	Reduction in ODI, VAS scores	Industry sponsored
De vivo et al. [18]	2020	Neuroradiology	Prospective uncontrolled trial	56	3, 12 months	Pain score (VAS), ODI	Reduction in VAS, ODI scores in 54/56 subjects	CT-guided technique
Fischgrund et al. [19]	2020	European Spine Journal	Prospective randomized double-blind sham-controlled multi-center	100	5+ years	ODI	Reduction in ODI scores	Industry sponsored
Macadaeg et al. [20]	2020	North American Spine Society Journal	Prospective open label single arm	48	6 weeks; 3, 6, 9, 12 months	ODI	Reduction in ODI, VAS scores	Industry sponsored
Smuck et al. [21]	2021	Regional Anesthesia and Pain Medicine	Prospective open label randomized controlled trial	140	6 weeks; 3, 6, 9, 12 months	ODI	Reduction in ODI scores	BVNA (*n* = 66) Standard care (*n* = 74) Industry sponsored
Koreckij et al. [22]	2021	North American Spine Society Journal	Prospective open label single arm randomized multi-center	140	2 years	Pain score (VAS), ODI	Reduction in ODI, VAS scores	Industry sponsored
Sherwood et al. [23]	2022	Pain Medicine	Retrospective review	N/A	N/A	N/A	Prevalence of BVNA eligibility was 11/338 (~3%) patient records	N/A
McCormick et al. [24]	2022	Pain Medicine	Pooled cohort	292	N/A	N/A	No specific MRI characteristic was strongly predictive of treatment outcome reliably	Facet fluid on MRI, weakly predictive of treatment failureIndustry sponsored
Boody et al. [25]	2022	Pain Medicine	Pooled cohort	292	N/A	N/A	No demographic or clinical characteristic that affected treatment outcome reliably	Industry sponsored
McCormick et al. [26]	2022	Pain Medicine	Pooled cohort	290	3 months	Pain score (VAS), ODI	Midline low back pain correlates with BVNA treatment success	Industry sponsored
Smuck et al. [27]	2023	Interventional Pain Medicine	Pooled cohort	95	3 years	ODI	Reduction in ODI, VAS scores	Industry sponsored
McCormick et al. [5]	2024	Pain Medicine	Pooled cohort	247205	15+ years	LBP-related healthcare utilization	Reduction in conservative treatment, opioid use, and lumbosacral injections	Industry sponsored
Smuck et al. [5]	2024	The Spine Journal	Cost–effectiveness analysis	N/A	N/A	N/A	>99% probability of cost-effectiveness in US of BVNA vs. standard care	Industry sponsored

VAS—Visual Analog Scale, ODI—Oswestry Disability Index. N/A, Not applicable.

## Data Availability

Data collected for this review are available from the corresponding author upon reasonable request.
